# Prevalence and associated factors of chronic non-communicable diseases among cross-country truck drivers in Ethiopia

**DOI:** 10.1186/s12889-020-09646-w

**Published:** 2020-10-17

**Authors:** Tewodros Yosef

**Affiliations:** Department of Public Health, College of Medicine and Health Sciences, Mizan-Tepi University, Mizan Teferi, Ethiopia

**Keywords:** Prevalence, Non-communicable diseases, Cross-country, Truck drivers, Ethiopia

## Abstract

**Background:**

Non-communicable diseases (NCDs) are diseases that are not transmitted from one person to another. Currently, NCDs are the primary causes of morbidity and mortality globally. Truck driving is an occupation that prone drivers to risk factors for NCDs than other occupations. Eventhough risk of developing NCDs among these population is high, studies that showed the prevalence and associated factors of chronic NCDs among truck drivers in Ethiopia are not available. Therefore, this study aimed to assess the prevalence and associated factors of chronic NCDs among truck drivers in Ethiopia.

**Methods:**

A cross-sectional study was conducted among 422 cross-country truck drivers at the Modjo dry port in Ethiopia. The interviwer-administered questionnaire technique was used to collect the data. The body mass index of the study participants was measured using DHM-15A standardized scale (BMI Height and Weight body fat scale).

**Results:**

Of the 400 truck drivers interviewed, the prevalence of chronic non-communicable diseases was 28.5, 95% CI (24.1–32.9%). Eighty (20%) had hypertension followed by 32(8%), and 22 (5.5%) had diabetes mellitus and asthma, respectively. The study also found that being married (AOR = 3.14, 95%CI [1.78–5.86]) and Separated/Divorced/Widower (AOR = 2.31, 95% CI [1.12–3.55]), having 3 or more family sizes (AOR = 1.46, 95% CI [1.33–4.42]), BMI ≥ 25 (AOR = 4.66, 95% CI [2.85–7.62]), smoking cigarettes [AOR = 1.71, 95% CI [1.03–2.81]), driving 10 or more years (AOR = 3.48, 95% CI [1.89–5.24]) and driving 9 or more hours daily (AOR = 3.76, 95% CI [1.96–6.54]) were statistically associated with chronic non-communicable diseases.

**Conclusion:**

The prevalence of chronic NCDs among truck drivers was significant (28.5%), and we can conclude that chronic NCDs are of public health importance among truck drivers in Ethiopia. This may create a substantial load on the healthcare system as an end result of increased demand and contact with healthcare services. Therefore, a rigorous effort is needed to develop strategies for the prevention and management of NCDs.

## Background

Non-communicable diseases (NCDs) are diseases that are not transmitted from one person to another, including cardiovascular diseases, cancer, chronic respiratory diseases, and diabetes. Currently, NCDs are the primary causes of disease and death globally [1, 2]. The magnitude of recent deaths from NCD sources alone is exceeding all other causes combined. They are anticipated to rise from 38 million in 2012 to 52 million by 2030 [2, 3]. The number of these deaths in low- and middle-income countries accounts for 80% and more than 90% of early deaths (deaths before the age of 70 years) happened in these countries [2, 4]. The prevalence of NCDs has increased gradually from time to time, which causes about 60% of disability-adjusted life years (DALYs) and 39.8 million deaths in 2015 alone universally [5].

In sub-Saharan Africa (SSA), NCDs cause a huge and rising burden of morbidity and mortality [6–9]. Non-communicable diseases are set to overtake communicable, maternal, neonatal, and nutritional (CMNN) diseases combined as the primary cause of death in SSA by 2030 [3]. The incidence and prevalence of NCDs are become rising in Ethiopia [10] and are mentioned as a cause for 42% of deaths (27% are early deaths before 70 years of age). The disability-adjusted life years increased from fewer than 20% in 1990 to 69% in 2015. Ethiopia is one of the SSA countries, which will experience the striking burden of early deaths and disability from NCDs by 2040 if no action is taken to avert the problem [11].

Occupation and work culture influence the lifestyle and related risk factors for NCDs [12]. Truck driving is an occupation that prone drivers to risk factors for NCDs than other occupations [13–16]. Truck drivers are among those professionals whose job and health status greatly influences public safety. Any health problem that affect drivers may result in an increased risk of road accidents [17]. Drivers may accidentally be affected by cardiovascular and cerebrovascular complications if hypertension and diabetes are not appropriately managed and can result in fatal road accidents if they occur while driving [18].

Apart from the role of genetics, the main risk factors for non-communicable diseases are behavioral; they relate to unhealthy diets such as diets low in fruits and vegetables, low in nuts and seeds, low in iron, and high in sodium are considerably connected with the incidence of NCDs [19]. Drivers are at greater warning of developing NCDs owing to an unhealthy diet, obesity, tobacco, harmful use of alcohol, and physical inactivity [20, 21]. Non-communicable diseases (NCDs) are the most important public health challenges that can be ward off through the prevention and control of behavioral risk factors [1, 22].

Truck drivers play a crucial role in the Ethiopian economy because of the limited rail, water, and other forms of transport of goods. The work-related nature of truck driving affects drivers to sedentary life and consequences in NCDs. Studies that showed the prevalence and associated factors of chronic NCDs among truck drivers in Ethiopia are not available. Designing appropriate intervention to respond to the problem, required sufficient data on the issue. Therefore, this study aimed to assess the prevalence and associated factors of chronic NCDs among truck drivers in Ethiopia.

## Methods

### Study design, area, and period

A cross-sectional study was conducted from February to March 2018 at the Modjo dry port. The port was the first dry port expansion established at the end of 2009 to get rid of the overcrowding of the Djibouti port. It is found in central Ethiopia, 38 miles southeast of Addis Ababa. The port handles 95% of Ethiopia’s trade and the major bottleneck in the Ethiopian-Djibouti trade passage [23].

### Populations

All cross-country truck drivers who drive between the Modjo dry port in Ethiopia and Djibouti international port were the source population. Those truck drivers who were systematically selected for the study were the study population.

### Study variables

The dependent variable was chronic non-communicable diseases (at least one of the Hypertension/Diabetes/Asthma). The independent variables were sociodemographic factors (age, educational status, monthly income, marital status, family size, body mass index), behavioral factors (smoking cigarettes, chewing chats, drinking alcohol, and physical activity), and occupational and psychosocial factors (average daily driving hours, total years of truck driving, rest breaks between driving, and perceived job stress).

### Sample size determination

A single population proportion formula was used to determine the sample size with an assumption of estimated proportion truck drivers with non-communicable diseases of 50% (Because of no previous study done regarding the prevalence of chronic non-communicable diseases among truck drivers in Ethiopia), 95% confidence interval with 5% precision level (with the expectation of the statistic value found within 5 percentage points of the real population value 95% of the time), and 10% for non-response compensation. The final computed sample size was 422.

### Sampling method

The potential participants were selected using a systematic random sampling method. In the absence of technical and accidental problem, a truck driver expected to make a travel from the Modjo dry port to the Djibouti port and back to Modjo dry port within 15 days. Based on the Modjo Dry Port Authority, an average of 300 to 400 trucks reaches daily at the port (Modjo Dry Port Authority report, 2017: Unpublished). Considerationing this, first, the determined sample size (422 truck drivers) was divided by 15 days and to interview approximately 28 drivers daily (422/15 = 28), and then the minimum daily truck flow was taken to be on the safe side, which was 300. Then 300 dividing by 28 to got skipping interval, which was 11. Finally, every eleventh driver from a random start was studied until the required sample size obtained.

### Operational definitions

**Prevalence**: the frequency of study subjects who were reported to have confirmed chronic non-communicable disease (at least one of Hypertension/Diabetes/Asthma) and on medication.

**A cigarette smoker** was a person who smoked cigarettes daily whatever the number of cigarettes [23, 24].

**An alcohol drinker** was a person who drinks (beer, local beer or areke, tella, or tej) every day or every other day [23].

**A chat chewer** was a person who chewing chats at least once within a week [23].

**Physically active**: a driver having 3 or more days (at least 30 min per day) of physical activity (walking, running, bicycling, and stretching exercises such as sit-ups and pull-ups) per week [23].

**Proper mealtime**: a driver taken his breakfast (7–9 am), lunch (12 am - 2 pm), and dinner (6 pm - 8 pm) based on the Ethiopian mealtime context [24].

**Rest break between driving**: a driver who takes rest after an hour or more of driving but not included rest for the meal [23].

### Data collection instrument and procedures

The interviwer-administered questionnaire technique was used to collect the data. The questionnaire was prepared after revising related literature, and it composed of socio-demographic, lifestyle, medical (NCD) information, occupational, ergonometric, and psych-social factors (Additional file 1- Questionnaire). First, it was prepared in English and then translated to the Amharic language, and then re-translated into English to maintain its consistencies. Pretest was done in 5% of the sample size in Akaki area, in which many trucks parked there. The body mass index of the study participants was measured using DHM-15A standardized scale (BMI Height and Weight body fat scale).

### Data processing and analysis

The collected data were processed and analyzed using statistical software. First, the data were entered into EPI-data version 4.2.0.0 and then analyzed using SPSS version 20 statistical software. A binary logistic regression model was computed. In the bivariate analysis, variables found significant at a *p*-value < 0.25 were eligible for multivariable analysis. In the Multivariable analysis model, independent variables found associated with the outcome of interest at a p-value < 0.05 were declared significant. Multi-collinearity between independent variables in the model was checked, and the variance inflation factor (VIF) was found acceptable (less than 2). The Hosmer-Lemeshow goodness-of-fit test indicated (*P* = 0.475) that the model was good enough to fit the data well.

## Results

### Socio-demographic characteristics

Out of 422 male truck drivers, 400 took part in this study, with a 94.8% response rate. The reason for non-response (5.2%) was the need for the incentive by respondents, as a result of the previous study by NGO on HIV prevalence had an incentive (5 USD for having HIV testing), some respondents think that the study had no any importance for them and some were not volunteered without any reason.

The mean age of respondents was 37.7 (±9.13 SD) years, range from 22 to 59 years. Ninety-six (24%) were single, and 41 (10.2%) reached college and university. The mean monthly income of drivers was 220 (±91) USD, range from 74 to 741 USD. Nearly two-thirds (66.5%) had 3 or more family sizes. Two hundred twenty-six (56.5%) had a BMI ≥ 25 kg/m^2^ (Table 1).

**Table 1:** Sociodemographic characteristics of the respondents at Modjo dry port in Ethiopia

Variables	Categories	Frequency (n)	Percent (%)
Age (years)	37.7 (±9.13) years		
Marital status	Single	96	24.0
	Married	268	67.0
	Separated/divorced/widower	36	9.0
Educational status	Read and write up to grade 8	92	23.0
	Grade 9–12	267	66.8
	College or University	41	10.2
Monthly income	< 220 USD	204	51.0
	≥ 220 USD	196	49.0
Family size	< 3	134	33.5
	≥ 3	266	66.5
BMI category (kg/m^2^)	18.5–24.9	174	43.5
	≥ 25	226	56.5

### Behavioral, occupational, and psychosocial factors

The proportion of shared behavioral risk factors among truck drivers was more prevalent. Of the 400 truck drivers interviewed, alcohol drinking was the most frequently reported shared behavioral risk factor, which accounted for 66%. The 63.8, 34.8, 31, and 48% of prevalence also reported physical inactivity, chewing chat, smoking cigarettes, and improper mealtime respectively. Two hundred four (51%) had six or more hours of sleep at night. Two hundred twenty-one (52.8%) of the drivers had fewer than 10 years of driving a truck. More than three-fourths (80.2%) reported nine or more hours on a typical day. Nearly three-fourths (75.8%) reported perceived job stress (Table 2).

**Table 2:** Behavioral, occupational, and psychosocial factors of the respondents at Modjo dry port in Ethiopia

Variables	Categories	Frequency (n)	Percent (%)
Smoking cigarette	Yes	124	31.0
	No	276	69.0
Alcohol drinking	Yes	264	66.0
	No	136	34.0
Chat chewing	Yes	139	34.8
	No	261	65.2
Physical activity	Yes	145	36.2
	No	255	63.8
Mealtime	Proper	208	52
	Improper	192	48
Hours took for sleeping at night	< 6 h	196	49.0
	≥ 6 h	204	51.0
Years of truck driving experience	< 10 years	221	52.8
	≥ 10 years	189	47.2
Average daily driving hours	< 9 h	79	19.8
	≥ 9 h	321	80.2
Rest break between driving	Present	339	84.8
	Absent	61	15.2
Perceived job stress	Yes	303	75.8
	No	97	24.2

### Prevalence of self-reported chronic non-communicable diseases

**Fig. 1:**
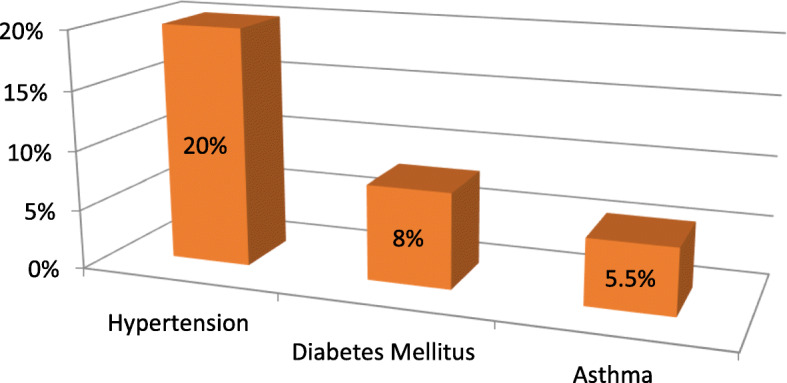
Types of chronic non-communicable diseases among respondents at Modjo dry port in Ethiopia

Of the 400 truck drivers interviewed, 28.5% (114) reported at least one of the confirmed chronic non-communicable diseases such as hypertension, diabetes mellitus, and asthma. Of the 400 truck drivers interviewed, hypertension was the most prevalent chronic NCDs accounted for 20% followed by 8 and 5.5% had diabetes mellitus (DM), and asthma, respectively, (Fig. 1).

### Factors associated with chronic non-communicable diseases

Bivariate and multivariable analysis were done to determine the association between dependent and independent variables. Finally, being married (AOR = 3.14, 95% CI [1.78–5.86]) and separated/divorced/widower (AOR = 2.31, 95% CI (1.12–3.55]), having three or more family sizes (AOR = 1.46, 95% CI (1.33–4.42]), BMI ≥ 25 (AOR = 4.66, 95% CI [2.85–7.62]), smoking cigarettes (AOR = 1.71, 95% CI [1.03–2.81]), driving 10 or more years (AOR = 3.48, 95% CI [1.89–5.24]), and driving 9 or more hours daily (AOR = 3.76, 95% CI [1.96–6.54]) were statistically associated with chronic non-communicable diseases (Table 3).

**Table 3:** Factors associated with chronic non-communicable diseases among respondents at Modjo dry port in Ethiopia

Variables	Categories	Non-Communicable Diseases		COR (95% CI)	AOR (95% CI)	P-value
		Yes	No			
Marital status	Single	11	85	1	1	
	Married	94	174	4.17 (2.76–6.12)**	3.24 (1.78–5.86)	**0.003**
	S/D/W^a^	9	27	2.58 (1.62–4.25)**	2.31 (1.12–3.55)	**0.018**
Family size	< 3	53	169	1	1	
	≥ 3	61	117	1.66 (1.09–5.58)**	1.46 (1.33–4.42)	**0.009**
BMI category (kg/m^2^)	18.5–24.9	14	167	1	1	
	≥ 25	100	119	10.2 (3.22–12.5)**	4.66 (2.85–7.62)	**< 0.001**
Smoking cigarette	Yes	46	78	1.80 (1.22–2.89)**	1.71 (1.03–2.81)	**0.033**
	No	68	208	1	1	
Physical activity	Yes	37	108	1	1	
	No	77	178	1.26 (0.89–2.14)*	1.08 (0.68–1.85)	0.523
Rest break between driving	Yes	106	233	1	1	
	No	8	53	0.33 (0.21–1.21)*	0.25 (0.23–1.09)	0.113
Years of driving experience	< 10 years	33	178	1	1	
	≥ 10 years	81	108	4.05 (2.52–6.43)**	3.48 (1.89–5.24)	**0.005**
Average daily driving hours	< 9 h	11	83	1	1	
	≥ 9 h	103	203	3.83 (2.10–7.02)**	3.76 (1.96–6.54)	**0.002**
Perceived job stress	Yes	91	212	1.38 (0.92–2.52)*	1.27 (0.76–2.37)	0.123
	No	23	74	1	1	

^a^Separated/Divorced/Widower

*CI* Confidence Interval, *COR* Crude odds ratio, *AOR* Adjusted odds ratio

*= significant at a *p*-value < 0.25

**= significant at a *p*-value < 0.05

## Discussion

The lifestyle and associated risk factors for NCDs greatly influnced by the Job and labor culture [12]. Truck driving is a job that lying drivers to risk factors for NCDs than other jobs [13–16]. Based on the above facts, this study aimed to assess the prevalence and associated factors of non-communicable diseases among cross-country truck drivers in Ethiopia. The overall prevalence of non-communicable diseases was 28.5, 95% CI (24.1–32.9%). This result was in line with 29.7% in the southern region of Ethiopia [25]. It was higher than 1.7% in northwest Ethiopia [26]. The prevalence of hypertension was 20, 95% CI (16.1–23.9%). This result was consistent with 18.86% in India [13], and 16.4% in Iran [17]. It was lower than 33.3% in Egypt [27], 27.7 and 33.1% in Nigeria [18, 28], 28.9 and 40% in India [15, 29], and 45.2% in Brazil [14]. However, it was higher than 10.5 and 16% in Ethiopia [11, 30].

The prevalence of diabetes was 8, 95% CI (5.3–10.7%). This result was consistent with 5.9 and 10.5% in Ethiopia [11, 30] and 9.1 in Iran [17]. It was lower than 12.2 and 13.1% in Ethiopia [25, 30]. However, it was higher than 3.4% in Nigeria [18] and 4.9% in Ethiopia [26]. The prevalence of asthma was 5.5, 95% CI (3.3–7.7%). This study was in line with 3.5 and 5.9% in Ethiopia [25, 31]. It was lower than 27.7% in Ethiopia [26]. But it was higher than 1.9% of asthma prevalence in Cameroon [32]. The variation observed compared to other studies could be owing to the differences in methodology, sample size, and operational definitions used. Besides, the socioeconomic, behavioral/lifestyle and cultural and educational profiles may create a significant variation.

Two-thirds (67%) and less than one-tenths (9%) were married and separated/divorced/widower respectively. Truck drivers who were married and separated/divorced/widower were 3.2 and 2.3 times, respectively more likely to develop NCDs than who never married. This study was consistent with studies conducted in Kenya and India [5, 33]. Approximately two-thirds (66.5%) had 3 or more family sizes. Those drivers with 3 or more family sizes were 1.5 times more likely to develop NCDs than drivers with fewer than 3 family sizes. Family size was strongly associated with the presence of NCDs. This study was supported by a study done in India [34].

Two hundred twenty-six (56.5%) were fall in 25 and above BMI category. Drivers with a BMI of 25 and above were 4.7 times increased odds of developing NCDs than those with normal BMI. BMI was strongly associated with NCD occurrence. This study was consistent with previous studies conducted elsewhere [10, 14, 15, 18, 20, 33–35]. Nearly one-thirds (31%) were smokers of cigarettes. The odds of having NCDs among smokers compared to nonsmokers were 1.7 times. This result was consistent with different studies conducted globally [12, 20, 33, 35].

One hundred eighty-nine (47.2%) had 10 or more years of driving. Drivers with 10 or more years of driving were 3.5 times more likely to have NCDs than those with fewer than 10 years. This could be due to the higher odds of having overweight/obesity associated with more years of driving. More than three-fourth (80.2%) of the respondents were driving for 9 or more hours within 24 h. Drivers with total daily driving of 9 or more hours were 3.8 times increased odds of developing NCDs than driving fewer than 9 h. The longer duration of diving hours creates more hours of sitting while driving resulted in overweight and obesity [15].

## Conclusion

The prevalence of chronic NCDs among truck drivers was significant (28.5%), and we can conclude that chronic NCDs are of public health importance among truck drivers in Ethiopia. This may create a substantial load on the healthcare system as an end result of increased demand and contact with healthcare services. Therefore, a rigorous effort is needed to develop strategies for the prevention and management of NCDs.

### Limitations

This study did not measure the blood pressure and blood glucose of study participants; rather only depend on self-reported data. Measuring the above-mentioned parameters may be important for getting the real prevalence and whether the blood pressure and blood glucose levels are well controlled or not. Besides, this study considers only major non-communicable diseases (hypertension, diabetes, and asthma) and leaves off other NCDs.

## Supplementary information

**Additional file 1.** Questionnaire

## Data Availability

The datasets used and/or analyzed during the current study are available from the corresponding author upon reasonable request.

## Abbreviations

AOR: Adjusted Odds Ratio
BMI: Body Mass Index
CI: Confidence Interval
COR: Crude Odds Ratio
DM: Diabetes Mellitus
HTN: Hypertension
NCDs: Non-communicable Diseases
SPSS: Statistical Package for the Social Sciences
SD: Standard Deviation
