# Supplementary material for: Prevalence and associated factors of chronic non-communicable diseases among cross-country truck drivers in Ethiopia
Source: BMC Public Health. 2020 Oct 17;20:1564. doi: 10.1186/s12889-020-09646-w (PMC7568414; doi:10.1186/s12889-020-09646-w)
Supplement: Supplementary file 1 — Additional file 1. Questionnaire [file 12889_2020_9646_MOESM1_ESM.docx]

**English Version Questionnaire Used to Assess NCDs among Truck Drivers**

Hello! My name is ___________. I am a data collector for carrying out a study on “Prevalence and Associated Factors of Chronic Non-Communicable Diseases among Cross-Country Truck Drivers in Ethiopia”. The result will provide important information on the ways to reduce the problem. Participating in this study will not have any risk or harm. You either have the full right to participate or decline participation in this study as a participant. You may respond to all the questions or you may not answer to questions you do not want to and you may end the interview at any time you want. You can ask any question that is not clear for you. Any information forwarded will be kept confidential and names will not be written or specified. The interview will take about 20 to30 minutes.

Now, are you willing to participate in this study?

1- No (say thank you) 2- Yes (continue interviewing)

If you have any question regarding the study, please contact Tewodros Yosef:

Cellphone: 0920478370 Email: tewodrosyosef47@mtu.edu.et

| **Part I: Socio-demographic information** | | | |
| --- | --- | --- | --- |
| **S.N.** | **Questions** | **Responses** | **Remark** |
| 101 | How old are you? | ______ in completed years |  |
| 102 | What is your religion? | 1. Protestant 2. Orthodox 3. Muslim 4. Others (specify)______ |  |
| 103 | What is your marital status? | 1. Single 2. Married 3. Divorced/separated 4. Widowed |  |
| 104 | What level of education did you achieve? | 1. Read & write up to Grade 8 2. Grade 9-12 3. College and university |  |
| 105 | Average monthly income in Ethiopian Birr (ETB) | ____________ |  |
| 106 | Weight in kg (measure) | **____________** |  |
| 107 | Height in cm (measure) | **____________** |  |

| **Part II: Lifestyle factors** | | | |
| --- | --- | --- | --- |
| 201 | Have you ever smoked cigarettes? | 1. Yes 2. No |  |
| 202 | If yes, have you smoked daily? | 1. Yes 2. No |  |
| 203 | Have you ever consumed alcohol (beer, areke, tella, tej)? | 1. Yes 2. No |  |
| 204 | If yes, how often? | 1. Every day or every other day 2. Once or twice in a week 3. 1-3 times in a month 4. Occasionally, less than once in a month |  |
| 205 | Have you ever consumed alcohol (beer, areke, tella, tej) two hours before driving or at the time of driving to keep awake while driving in the last month? | 1. Yes 2. No |  |
| 206 | Have you ever chewed chat? | 1. Yes 2. No |  |
| 207 | If yes, how often? | 1. Every day or nearly every day 2. Once or twice a week 3. 1-3 times a month |  |
| 208 | Have you ever chewed chat two hours before driving or at the time of driving to keep awake while driving in the last month? | 1. Yes 2. No |  |
| 209 | Have you ever had physical activity at least 30 minutes a day? | 1. Yes 2. No |  |
| 210 | If yes, how often? | 1. < 3 days 2. ≥ 3 days |  |
| 211 | Have you had breakfast (7-9 am), lunch (12 am- 2 pm), and dinner (6 pm-8 pm) time? | 1. Yes 2. No |  |
| 212 | How many hours spent sleeping? | __________ |  |
| **Part III: Medical information** | | | |
| 301 | Have you confirmed diagnosis of non-communicable diseases? | 1. Yes 2. No |  |
| 302 | If yes, what type of non-communicable disease? (multiple answers possible) | 1. Hypertension 2. Diabetes 3. Asthma |  |
| 303 | How many years since diagnosed and treated? | _________ |  |
| 304 | Are you currently on treatment? | 1. Yes 2. No |  |
| 306 | Are you satisfied with the treatment you took? | 1. Yes 2. No |  |
| 307 | Do you have a family history of at least one of the above mentioned (Q 402) non-communicable diseases? | 1. Yes 2. No |  |
| **Part IV: Occupational, Ergonometric and Psych-social factors** | | | |
| 401 | How many years spent on truck driving? | __________ |  |
| 402 | How many hours do you drive in a day? | __________ |  |
| 403 | Do you have a rest break in between driving? | 1. Yes 2. No |  |
| 404 | If yes, how many hours? | __________ |  |
| 405 | Does your former job require prolonged sitting? | 1. Yes 2. No |  |
| 406 | Do you think the job is stressful? | 1. Yes 2. No |  |
| 407 | Have you felt boredom with driving? | 1. Yes 2. No |  |
| 408 | Do you think you have enough time to spend with your family? | 1. Yes 2. No |  |

**Thank you for your cooperation**
